# The publication fate of abstracts presented at the Medical Library Association conferences

**DOI:** 10.5195/jmla.2021.1220

**Published:** 2021-10-01

**Authors:** Rachel J. Hinrichs, Mirian Ramirez, Mahasin Ameen

**Affiliations:** 1 rhinrich@iupui.edu, Health Sciences Librarian, IUPUI University Library, Indiana University Purdue University Indianapolis, Indianapolis, IN; 2 mirirami@iu.edu, Research Metrics Librarian, Ruth Lilly Medical Library, Indiana University, Indianapolis, IN; 3 mameen@iupui.edu, Teaching and Learning Librarian, IUPUI University Library, Indiana University Purdue University Indianapolis, Indianapolis, IN

**Keywords:** publishing, congresses as topic, libraries, medical, health sciences librarians

## Abstract

**Objective:**

We sought to determine how many abstracts presented at the 2012 and 2014 Medical Library Association (MLA) annual conferences were later published as full-text journal articles and which features of the abstract and first author influence the likelihood of future publication. To do so, we replicated a previous study on MLA conference abstracts presented in 2002 and 2003. The secondary objective was to compare the publication rates between the prior and current study.

**Methods:**

Presentations and posters delivered at the 2012 and 2014 MLA meetings were coded to identify factors associated with publication. Postconference publication of abstracts as journal articles was determined using a literature search and survey sent to first authors. Chi-squared tests were used to assess differences in the publication rate, and logistic regression was used to assess the influence of abstract factors on publication.

**Results:**

The combined publication rate for the 2012 and 2014 meetings was 21.8% (137/628 abstracts), which is a statistically significant decrease compared to the previously reported rate for 2002 and 2003 (27.6%, 122/442 abstracts). The odds that an abstract would later be published as a journal article increased if the abstract was multi-institutional or if it was research, specifically surveys or mixed methods research.

**Conclusions:**

The lower publication rate of MLA conference abstracts may be due to an increased number of program or nonresearch abstracts that were accepted or a more competitive peer review process for journals. MLA could increase the publication rate by encouraging and enabling multi-institutional research projects among its members.

## INTRODUCTION

Librarians attend a wide array of professional conferences designed to encourage networking, “professional rejuvenation,” and knowledge sharing [1]. Formal and informal knowledge sharing plays a significant role in the translation of evidence from research to practice. A scoping review of conference objectives and evaluations found that conference planners frequently cite knowledge acquisition, transfer, and research dissemination as their primary objectives [2]. While conferences are useful platforms to share information, posters and presentations do not enable the full dissemination of details and do not undergo rigorous peer review like journal articles. There is also the issue of individuals in the profession who are unable to attend conferences. How will they stay up to date on the latest trends and practices of the discipline? Even if abstracts are published online for free, the evidence could be considered “lost” if there is not enough detail to implement the findings into practice. To enable the full dissemination of details, conference presenters are encouraged to share their work more broadly through a full-text publication. Subsequent publications extend the conversations taking place at conferences and increase their impact on the profession [3]. The publication rate of conference abstracts to full-text articles is the most utilized indicator of success of conferences [2]. In fact, a “conference impact factor” has been proposed based on the number of published articles resulting from a conference and the impact factors of the journals in which they were published [4].

Despite the benefits of publication, more than half of abstracts presented at health and medical conferences are not published [5]. A Cochrane review of 425 conference abstract studies found that only 37.3% of abstracts were later published [5]. The publication rate of abstracts presented at library and information science (LIS) conferences has been consistently lower. This ranges from 13% at the Association of College and Research Libraries (ACRL) conference in 1999 to 32% at The International Society for Scientometrics and Informetrics [6, 7]. The publication rate for medical library conferences appears to be in-between—28% for Medical Library Association (MLA) conferences in 2002 and 2003 and 32% for Canadian Health Libraries Association (CHLA) conferences between 2004 and 2009 [8, 9].

Many factors contribute to authors' decisions on whether or not to turn a conference presentation into a full-text article. A systematic review of medical and health care research found that time was the most frequently reported reason, followed by lack of resources, publication not a goal, low priority, and trouble with coauthors [5]. These reasons are similar to those reported in the LIS literature; time and not intended for publication were the primary reasons reported by presenters at MLA and CHLA conferences [8, 9]. Authors at these conferences also reported that they did not publish because their work was not substantial enough [8, 9]. Compared to scientific conferences, LIS conferences are practice-based and more often feature successful projects, best practices, or technical content that the authors may feel are not suited for publication [6–9]. The conferences analyzed by Cochrane were primarily research based [5]. If only LIS research abstracts are considered, as done by Alpi and colleagues in their study of award-winning research abstracts presented at MLA, the publication rate appears to be higher at 37%, which is more in line with the Cochrane review [5, 10]. This could be because librarians value research over nonresearch for publication. Harvey and Wandersee suggest that medical librarians may undervalue their potential contributions to the literature because they are overly aware of methodological limitations or fear rejection [8]. Shaw and Szwajcer refer to this issue as a “confidence gap” that results in an “unwillingness to engage in the publication process” [9]. In other cases, there could be a lack of organizational structure or incentives that encourage publication. A survey of MLA members found that librarians working in hospitals were less likely to present at a conference or publish a paper compared to academic health sciences librarians, especially those pursuing tenure [11]. Differences in who publishes and what gets published can result in publication bias and limit whose voices are heard. Ultimately, this bias could affect the evidence that practitioners rely on for practice.

With this in mind, the primary objective of this study was to determine which features of a conference abstract and first author influence the likelihood of future publication as a journal article. To do so, we replicated Harvey and Wandersee's study of the publication rate of MLA conference abstracts presented in 2002 and 2003 using the MLA abstracts from 2012 and 2014. We hypothesized that research abstracts were more likely to be published than nonresearch abstracts. The secondary objective was to compare publication rates between the present and previous studies [8].

## METHODS

### Abstract inclusion

This study is a modified replication of Harvey and Wandersee's original study of abstracts presented in 2002 and 2003 [8]. All abstracts for presentations and posters accepted for the 2012 and 2014 MLA conferences were included [12]. We selected these years because they gave authors at least five years to publish [5]. The MLA conference in 2013 was skipped because it was a joint conference with international librarian associations and included more sessions than a typical MLA conference. Lightning talks, tech trends, and invited presentations were excluded. Invited presentations were identified based on the description in the program and a list of invited speakers and presentation titles provided by MLA headquarters to the authors. Abstracts that only had a title were also excluded. This resulted in a total of 628 abstracts. Subsequent publication as a journal article was determined using two methods—a literature search and a survey of first authors. This study was reviewed by the Indiana University Institutional Review Board and was determined to be exempt (protocol number 1911002254).

### Abstract data extraction

Librarians at Indiana University Purdue University Indianapolis (IUPUI) (n=7) were recruited to read and extract data from each abstract. Reviewers all had MLS degrees and at least some familiarity with LIS research methods. Before reviewing began, two norming sessions were held to familiarize the reviewers with the study protocol, to practice extracting data from selected abstracts, and to norm the results. Our original plan was to have all 628 abstracts independently reviewed by two librarians, with any disagreements resolved during a consensus meeting. Due to time restraints, 176 abstracts (28%) were only extracted by one reviewer (RH); the remaining 452 abstracts (72%) were extracted by two reviewers.

Reviewers extracted the following data from each abstract into Qualtrics, a cloud-based survey tool: a unique ID, conference year, title, first author full name, format (poster or presentation), work setting of first author, AHIP status of first author, international (non–United States) institution, nonlibrarian author, and single-/multi-institutional. Work setting included college or university, hospital, government or health association library, and other. While some abstracts had coauthors that worked in different settings, we decided to record only the first author's work setting. Nonlibrarian authors included health care professionals, research and teaching faculty, clinicians, vendor representatives, programmers, web developers, and LIS faculty members. Librarian authors included informationists, library directors, library staff members, library school students, and library fellows.

A determination was also made about whether an abstract was research. We used a definition of research that had been used in two previous LIS research content analyses: an “inquiry which is carried out, at least to some degree, by a systematic method with the purpose of eliciting some new facts, concepts, or ideas” [13, 14]. If the abstract was determined to be research, the research method was also recorded (Appendix A). Whether an abstract was research was often difficult to determine with the limited information available in an abstract. Of the 452 abstracts reviewed by two people, we had disagreements on the research status for 60 abstracts (13%); an additional 30 abstracts (7.5%) we agreed were research but disagreed on the method used. All conflicts were resolved through a consensus meeting between the two reviewers. We calculated Cohen's kappa for inter-rater reliability for the abstracts that had been reviewed twice and found that agreement on the research status of the abstracts was 0.68, which is considered substantial agreement.

### Literature search for full-text journal articles

One reviewer conducted a search in Google Scholar, Library, Information Science & Technology Abstracts (LISTA), and PubMed in order to determine if an abstract had later been published as a journal article. Searches were conducted using the first author name in each database and then coauthors if no publication was found. If there were too many results to review, a relevant keyword was added. To determine a match, the full-text article needed to have at least one common author name, exact or closely related objectives, and exact or very similar study methods. Pilot studies and preliminary analyses were included. Publications with substantial changes in the study methods or objectives were excluded. If a match was found, the title of the journal, year and month of publication, and DOI, PMID, or URL of the article were recorded (Appendix B). Book chapters, dissertations, digital projects, blog posts, and white papers were excluded.

### Survey of first authors

A questionnaire was developed to survey the first authors of the abstracts. The purpose of the questionnaire was to identify published articles that were missed by the search, to provide a check for accuracy on the articles identified by the search, and to gather information on the author's formal credentials at the time of the abstract presentation.

Using the Qualtrics platform, we created and distributed a survey through an email message to each first author. We created a custom authors' email addresses list in Qualtrics to automatically send an email that included the questionnaire link and the titles of each abstract they presented. We were able to gather the email addresses of 434 out of 487 unique authors; 53 email addresses were not found, or the author was retired, had changed careers, or had passed away.

For this study, we used a questionnaire adapted from the Harvey and Wandersee survey [8]. Their survey required authors to only answer for the first abstract presented if they had presented multiple abstracts. However, we modified the survey to ask authors about all abstracts where they appeared as first authors. We provided the titles of the abstracts they presented in the email we sent and in the survey itself. The questionnaire consisted of several close-ended, open-ended, and multiple selection questions to collect answers about the submission stage, publication venue, peer review status of the article, year of publication, primary and secondary reasons to pursue the publication, and their credentials at the time of the conference (Appendix C).

An initial invitation to complete the survey was sent to the 434 first authors in August 2020. Reminder emails were sent only to unfinished recipients two weeks later and then one week after that. The survey was kept open for a month total.

### Data analysis

Data were analyzed using IBM SPSS Statistics (version 26). Counts and percentages were used to summarize nominal data. Publication rates were compared using the chi-square test. The influences of various factors on the odds that the abstract was subsequently published as a journal article was assessed using logistic regression with odds ratios (ORs) and 95% confidence intervals (CIs) being generated. The factors assessed were format, single-/multi-institutional, work setting, first author's AHIP status, international (non–United States) institution, nonlibrarian author, first author's highest credential, and research method. A p-value <0.05 was considered statistically significant. Reference levels were selected based on data end points and outcomes of interest.

## RESULTS

### Abstract characteristics and survey results

Data were extracted from a total of 628 abstracts presented at the 2012 and 2014 annual MLA conferences. Table 1 summarizes the abstract characteristics, and Table 2 summarizes the methods used by the research abstracts.

**Table 1:** Abstract characteristics (n=628)

Variable	Number of abstracts (%)
*Conference year*	
2012	325 (51.8)
2014	303 (48.2)
*Format*	
Poster	405 (64.5)
Presentation	223 (35.5)
*Work setting*	
University or college	523 (83.3)
Hospital	55 (8.7)
Government or health association	29 (4.6)
Other	21 (3.3)
*International (non-US)*	
No	578 (92.0)
Yes	50 (8.0)
*Nonlibrarian author*	
No	471 (75.0)
Yes	157 (25.0)
*Multi-institutional*	
No	495 (78.8)
Yes	133 (21.2)
*First author has AHIP credential*	
No	425 (67.7)
Yes	203 (32.3)
*Research-based*	
No	448 (71.3)
Yes	180 (28.7)
*First author highest credential*	
MLS	422 (67.2)
Non-MLS master's degree	112 (17.8)
PhD	55 (8.8)
Professional degree (MD, RN, etc.)	24 (3.8)
Not identified or reported in survey	13 (2.1)
Library school student	2 (0.3)
*Published as journal article*	
No	491 (78.2)
Yes	137 (21.8)

**Table 2:** Methods used in research abstracts (n=180)

Research method	Number of abstracts (%)
Survey	51 (28.3)
Mixed methods	32 (17.8)
Content analysis	21 (11.7)
Bibliometrics	18 (10.0)
Experimental	17 (9.4)
Focus groups/interviews	14 (7.8)
Observational or descriptive studies	12 (6.7)
Literature reviews	8 (4.4)
Secondary data analysis	7 (3.9)

The electronic survey was emailed to 434 first authors, and 34 emails bounced or failed. Of the 400 successful emails, 161 people responded to the survey for a response rate of 40.3%. Credentials for those who did not respond to the survey were determined through a Google search for their CV, ORCID profile (https://orcid.org/), or LinkedIn (https://www.linkedin.com/) profile.

### Full-text publication rate

Of the 628 abstracts, 137 abstracts (21.8%) were published as full-text journal articles. Most of these articles (n=132) were found via the literature search; the survey identified an additional five articles that the search had missed. This publication rate was significantly less than the 27.6% rate reported for 2002 and 2003 (*X*^2^=4.73, *p*=0.03) [8]. Notably, MLA also saw an increase in the number of abstracts presented, from a low point of 189 abstracts in 2002 to a high point of 325 abstracts in 2012. Twelve (1.9%) abstracts were published before the conference presentation. Although it is not clear whether Harvey and Wandersee included previously published abstracts in the 2002–2003 publication rate [8], we chose to include these 12 abstracts as published.

### Factors contributing to full-text publication

For the analysis of factors contributing to full-text publication, we excluded the 12 abstracts that had already been published. We also excluded an additional 15 abstracts due to missing author credential data (n=13) and an inadequate sample size for the author credential category “library school student” (n=2). As a result, 601 articles were included in the logistic regression analysis of factors contributing to full-text publication.

### Factors associated with authors

Factors associated with authors included the first author's highest credential, AHIP certification of first author, and whether there was a nonlibrarian coauthor. For the author credentials, we were interested in seeing if authors who have advanced degrees in addition to or other than an MLS degree were more likely to publish, as authors with non-MLS degrees may have more advanced research training than authors with only an MLS degree. It turned out that many authors had more than one advanced degree; in fact, 164 abstracts (26.1%) were presented by authors with two or more advanced degrees. In these cases, we selected the first author's highest credential in the following order: PhD > professional degree > non-MLS master's degree > MLS. However, none of these author-level factors were significantly associated with publication rate (Table 3).

**Table 3:** Potential variables associated with publication: authors

Variable	Published n (%)	Not published n (%)	OR (95% CI)	*p*-value
Nonlibrarian author	36 (24.7)	110 (75.3)	1.2 (0.73–2.0)	0.453
First author has AHIP credential	46 (23.4)	151 (76.6)	1.2 (0.76–1.9)	0.436
First author's highest credential				
MLS	83 (19.9)	334 (80.1)	REF	REF
Non-MLS master's degree	20 (18.5)	88 (81.5)	0.89 (0.50–1.6)	0.693
Professional degree (MD, RN, etc.)	3 (13.6)	19 (86.4)	0.62 (0.17–2.2)	0.462
PhD	16 (29.6)	38 (70.4)	1.3 (0.61–2.7)	0.527

REF = Reference Group

### Factors associated with setting or institution

Factors associated with the institution included the work setting, international (non–United States) institution, or single-/multi-institutional, all identified by the author affiliations. Of these, multi-institutional abstracts were 1.7 times more likely to be published than those from single institutions (Table 4). International institution and work setting were not significantly associated with publication rate.

**Table 4:** Potential variables associated with publication: setting or institution

Variable	Published n (%)	Not published n (%)	OR (95% CI)	*p*-value
Multi-institutional	35 (28.2)	89 (71.8)	1.7 (1.0–2.7)	0.046
International	13 (30.2)	30 (69.8)	1.1 (0.51–2.5)	0.749
Work setting				
University or college	105 (20.9)	397 (79.1)	REF	REF
Government or health association	2 (7.4)	25 (92.6)	0.23 (0.05–1.0)	0.057
Hospital	13 (24.5)	40 (75.5)	1.2 (0.61–2.5)	0.546
Other	2 (10.5)	17 (89.5)	0.38 (0.08–1.7)	0.208

REF = Reference Group

**Table 5:** Potential variables associated with publication: format and research method

Variable	Published n (%)	Not published n (%)	OR (95% CI)	*p*-value
Format				
Poster	74 (19.3)	309 (80.7)	REF	REF
Presentation	48 (22)	170 (78)	1.2 (0.75–1.8)	0.503
Research method				
Bibliometrics	5 (27.8)	13 (72.2)	2.2 (0.76–6.8)	0.144
Content analysis	4 (20)	16 (80)	1.2 (0.39–3.9)	0.715
Experimental	3 (20)	12 (80)	1.2 (0.32–4.5)	0.788
Focus groups/interviews	3 (21.4)	11 (78.6)	1.1 (.29–4.5)	0.846
Literature review	1 (12.5)	7 (87.5)	.58 (.07–5.1)	0.628
Mixed methods	16 (51.6)	15 (48.4)	4.9 (2.2–11)	<0.01
Observational/descriptive/field study	2 (16.7)	10 (83.3)	1.0 (0.21–5.0)	0.960
Secondary data analysis	2 (28.6)	5 (71.4)	2.5 (0.44–13.7)	0.302
Survey	15 (32.6)	31 (67.4)	2.3 (1.2–4.7)	0.015
Not research	71 (16.5)	359 (83.5)	REF	REF

REF = Reference Group

### Factors associated with format and research method of abstract

Full-text publication was not significantly associated with whether the abstract was a poster or presentation. Whether an abstract was research, however, was significantly associated with publication rate; 29.8% (n=51) of research abstracts were later published compared to only 16.5% (n=71) of nonresearch abstracts (*X*^2^=13.403, *p*<0.001). Mixed methods research and surveys were, respectively, 4.9 and 2.3 times more likely to get published than nonresearch abstracts.

### Full-text publication features

Articles were published in 36 unique journals. Three journals published 64% of the articles—*Medical Reference Services Quarterly* (n=35), the *Journal of the Medical Library Association* (n=27), and the *Journal of Hospital Librarianship* (n=24). These are the same top journals found in Harvey and Wandersee's study as well, which suggests that these journals are still highly relevant for health sciences librarians [8]. Eleven articles were published in non-LIS journals.

## DISCUSSION

### Full-text publication rate

Results from this study found that 21.8% of abstracts presented at the 2012 and 2014 MLA conferences are published as journal articles. This is lower than the 27.6% rate reported previously [8]. There are several possible reasons for this lower rate. One, an increase in the number of accepted abstracts that tend to not get published (i.e., nonresearch and single-institution abstracts) could lower the publication rate. A larger number of abstracts were presented at the 2012 and 2014 MLA conferences compared to the 2002 and 2003 conferences (628 versus 442), though it is unknown if those abstracts tended to be nonresearch or based at one institution because the previous study did not gather that data [8]. Despite an increase in the number of accepted abstracts, there may not be an increase in the number of authors willing to publish. In fact, the number of published articles from the previous study compared to ours is very close—123 versus 137 [8]. Another reason for the lower rate may be that scholarly journals' acceptance rates are more stringent or that there is a limit on the number of articles that the journals want to publish. A comparison of journal acceptance rates and number of articles published over time might lend some more insight into this. However, no authors in our survey reported that their manuscript was rejected.

Either way, more librarians are presenting their work at MLA. More presentations may mean that more people can attend the conference. Some institutions will not provide financial support to attend conferences unless the attendee is presenting [16]. Presenting may be seen as more attainable than publication; surveys consistently show that librarians are more likely to present at a conference than publish a paper [11, 15, 17]. Librarians may consider their conference presentation as the “final product” or, similarly, they may not consider a peer-reviewed article as their “primary desired research output” [6, 17, 18]. Beyond a journal article, librarians may choose to share their work in other ways, such as through blogs, social media, personal websites, newsletters, or institutional repositories. The survey from this study confirmed that two people later published a book chapter based on their conference abstract, and one person published their work in an e-newsletter. These other venues may allow librarians to share their work without as much of a time burden. It is also possible that these other formats seem more appropriate to report projects that are not research.

### Factors contributing to full-text publication

This study found that multi-institutional and research abstracts, specifically studies using mixed methods or surveys, were more likely to get published. An abstract with authors from more than one institution may imply that there is collaboration on a bigger project than could be done at a single institution. For example, the study could involve participants from different population groups, which could increase the generalizability of the results. These studies could be perceived as being more important and thus a better fit for a journal article. Many other studies have confirmed that multi-institutional studies are more likely to get published [5, 19, 20].

Whether an abstract was research was the strongest predictor of publication, which supports our hypothesis and confirms what Alpi and colleagues found in their study [10]. It is likely that a research abstract is more generalizable and easier to translate to a journal article. The author may have even developed the study with the intention of publication. Our findings show that surveys and mixed methods, a combination of qualitative and quantitative methods, were the most commonly used research methods and also the most likely to be published compared to nonresearch abstracts. Surveys are consistently the most common research method used in LIS journal literature [14, 21–23].

Surprisingly, work setting was not a statistically significant factor, though the sample of hospital librarians was very small compared to academic librarians. Lessick and colleagues found that hospital librarians were less likely than other types of health sciences librarians to present at conferences, publish research, or engage in other research activities [11]. Myers found in her content analysis of MLA conference abstracts that hospital librarians presented approximately 18% of the abstracts, which is similar to the proportion of hospital librarian membership in MLA [15]. Our analysis found that only 8.7% of the abstracts were presented by hospital librarians as first authors. It is possible that this number would be higher if the coauthors' affiliations were considered. However, in this study, hospital librarians were not less likely than academic librarians to publish; 24.5% (n=13) of abstracts from hospital settings were later published as journal articles. Seven of those articles (53.8%) were published in the *Journal of Hospital Librarianship*, which confirms the importance of this journal for hospital librarians.

### Strengths and limitations

There are several strengths and limitations to this study. A major strength is the use of two different methods to determine the publication rate and to build a comprehensive dataset, which could be used for other research projects. Other strengths include a long follow-up to allow enough time for publication and the use of several different criteria to match abstracts to publications. One limitation is the sample of two conferences for the data. While the number of abstracts included is substantial, the results from these two conferences may not be generalizable to MLA conferences as a whole. MLA conferences are often theme based, which may lead to variation in the content and types of abstracts that get accepted for a given year. Having two years of conference data may have mitigated this limitation to some extent. Another limitation includes the use of only the first author's work setting, which may have underestimated hospital librarians' participation. Not all abstracts were reviewed by two people, which could have biased the results. Finally, it was often difficult to determine whether an abstract was research, and, if so, what method was used. Abstracts have limited information compared to a full article, and often MLA abstracts only include the objective and methods because the results were not complete at the time of submission. Reporting was poor or unclear in many abstracts. We highly recommend that individuals interested in submitting to any conference consider the use of abstract reporting guidelines, which can be found on the Equator Network website [24].

### Implications

Publication rate may be the most utilized indicator of success for a conference, but we believe that it is not be best indicator of success for LIS conferences [2]. Other studies of library conference abstracts express concern about the low rate of journal article publication and potential loss of information [6, 8, 9]. However, librarianship is a practice-based discipline, and we wonder if a better approach would be to consider ways to improve the dissemination of successful programs, best practices, and nonresearch literature. Librarians are understandably selective in determining whether an abstract is “worthy” of being developed into a journal article, which takes a significant amount of time and investment. Instead of writing a journal article, librarians could write a shorter, practice-focused article for newsletters such as *MLAConnect* (https://www.mlanet.org/mlaconnect). Alternatively, they could deposit their poster in an institutional repository or a personal website to make it more easily discoverable online. MLA could consider awarding the most innovative program abstracts at each conference, similar to what is done for research abstracts. Program and nonresearch abstracts can offer personal experiences and authors' lived expertise, which can be greatly valuable to other practitioners.

On the other hand, there were many abstracts that to us appeared to be good candidates for a journal article, but we were not able to locate a publication. How do we ensure that more research abstracts get published? MLA has several ongoing initiatives to increase librarians' research confidence and skills, but perhaps a more hands-on approach could be taken. Eldredge and colleagues tested an interesting approach called “real time peer review” where conference attendees offered “direct, immediate, and actionable feedback” to presenters, and selected presenters were mentored by a colleague who encouraged them to publish [25]. A similar approach could be taken for all abstracts that win a research or program award.

### Conclusions

While conferences promote knowledge sharing among attendees, full-text publications enable the full dissemination of findings and build an evidence base for a profession. We found that 21.8% of the abstracts presented at the 2012 and 2014 MLA conferences were published as journal articles. Presenters at both conferences published at a lower rate than observed in a past study. The lower publication rate may be due to an increased number of abstracts that are accepted or a more competitive peer review process among journals. Authors' decision to publish is influenced by many factors, including time and whether they believe their work is substantial enough to warrant publication. In our study, multi-institutional and research abstracts were more likely to get published. These types of abstracts may be seen as more substantial projects or as easier to translate to a journal article. MLA could increase the publication rate by enabling multi-institutional research projects among its members, or by taking a hands-on approach to encouraging members to publish such as “real time peer review” [25]. Librarians should also consider alternative ways to share their work outside of a journal article. Building a substantial evidence base requires librarians to not only engage with the research process, but to consider other ways of distributing best practices and program ideas beyond a conference presentation.

## Data Availability

Data associated with this article are available at IUPUI DataWorks at 10.7912/D2/19.

## SUPPLEMENTAL FILES

**Appendix A:** Research method definitions

**Appendix B:** Abstract data extraction instrument

**Appendix C:** Author questionnaire

## References

[1] Vega RD, Connell RS. Librarians' attitudes toward conferences: a study. Coll Res Libr. 2007;68(6):503–14. DOI: 10.5860/crl.68.6.503.

[2] Neves J, Lavis JN, Ranson MK. A scoping review about conference objectives and evaluative practices: how do we get more out of them? Health Res Policy Sys. 2012;10(1):26. DOI: 10.1186/1478-4505-10-26. PMC3487916 22857399

[3] González-Santos S, Dimond R. Medical and scientific conferences as sites of sociological interest: a review of the field. Sociol Compass. 2015;9(3):235–45. DOI: 10.1111/soc4.12250.

[4] Lang R, Porter K, Krentz HB, Gill MJ. Evaluating medical conferences: the emerging need for a quality metric. Scientometrics. 2020;122(1):759–64. DOI: 10.1007/s11192-019-03291-w.

[5] Scherer RW, Meerpohl JJ, Pfeifer N, Schmucker C, Schwarzer G, von Elm E. Full publication of results initially presented in abstracts. Cochrane Database Syst Rev. 2018;(11). DOI: 10.1002/14651858.MR000005.pub4. PMC7073270 30480762

[6] Fennewald J. Perished or published: the fate of presentations from the ninth ACRL conference. Coll Res Libr. 2005;66(6):517–26. Available from: http://crl.acrl.org/index.php/crl/article/view/15760.

[7] Aleixandre-Benavent R, González-Alcaide G, Miguel-Dasit A, Navarro-Molina C, Valderrama-Zurián J. Full-text publications in peer-reviewed journals derived from presentations at three ISSI conferences. Scientometrics. 2009;80(2):407–18. Available from: https://akjournals.com/view/journals/11192/80/2/article-p407.xml.

[8] Harvey SA, Wandersee JR. Publication rate of abstracts of papers and posters presented at Medical Library Association annual meetings. J Med Libr Assoc. 2010 Jul;98(3):250–5. DOI: 10.3163/1536-5050.98.3.014. 20648260 PMC2901010

[9] Shaw CE, Szwajcer AL. Publication rate of presentation abstracts presented at the Canadian Health Libraries Association (CHLA/ABSC) annual meetings from 2004-2009. Performance Measurement Metric. 2016;17(3):252–62. DOI: 10.1108/PMM-07-2016-0034.

[10] Alpi KM, Fenske R. Previous research shows Medical Library Association award winner publication rate. J Med Libr Assoc. 2012;99(1):3. DOI: 10.3163/1536-5050.99.1.002. PMC3016645 21243048

[11] Lessick S, Perryman C, Billman BL, Alpi KM, De Groote SL, Babin TD. Research engagement of health sciences librarians: a survey of research-related activities and attitudes. J Med Libr Assoc. 2016;104(2):166–73. DOI: 10.5195/jmla.2016.68. 27076808 PMC4816469

[12] MLA: Meetings : Past and future meetings [Internet]. Medical Library Association. [cited 2020 Nov 16]. Available from: https://www.mlanet.org/p/cm/ld/fid=56.

[13] Peritz BC. The methods of library science research: some results from a bibliometric survey. Library Research. 1980;2(3):251–68.

[14] Gore SA, Nordberg JM, Palmer LA, Piorun ME. Trends in health sciences library and information science research: an analysis of research publications in the Bulletin of the Medical Library Association and Journal of the Medical Library Association from 1991 to 2007. J Med Libr Assoc. 2009;97(3):203–11. Available from: https://www.ncbi.nlm.nih.gov/pmc/articles/PMC2706445/. 19626146 10.3163/1536-5050.97.3.009 PMC2706445

[15] Myers B. What we talk about when we talk about medical librarianship: an analysis of Medical Library Association annual meeting abstracts, 2001–2019. J Med Libr Assoc. 2020 Jul 1;108(3):364–77. DOI: 10.5195/jmla.2020.836. 32843868 PMC7441905

[16] Neville TM, Henry DB. Support for research and service in Florida academic libraries. J Acad Librariansh. 2007;33(1):76–93. DOI: 10.1016/j.acalib.2006.06.003.

[17] Hoffmann K, Berg S, Koufogiannakis D. Understanding factors that encourage research productivity for academic librarians. EBLIP. 2017;12(4):102–28. DOI: 10.18438/B8G66F.

[18] Drott MC. Reexamining the role of conference papers in scholarly communication. J Am Soc Inform Sci Tech. 1995;46:299–305. DOI: 10.1002/(SICI)1097-4571(199505)46:4<299::AID-ASI6>3.0.CO;2-0.

[19] Light A, Dadabhoy M, Burrows A, Nandakumar M, Gupta T, Karthikeyan S, Daniel A. Publication fate of abstracts presented at four British surgical meetings: an 11-year follow-up. J Surg Res. 2019;234:139–48. DOI: 10.1016/j.jss.2018.09.047. 30527466

[20] Spencer S, Majkowski C, Suda KJ. Predictors of publication rates for abstracts presented at the American Association of Colleges of Pharmacy annual meetings. Am J Pharm Educ. 2018;82(8):6409. DOI: 10.5688/ajpe6409. 30425404 PMC6221525

[21] Turcios ME, Agarwal NK, Watkins L. How much of library and information science literature qualifies as research? J Aca Librariansh. 2014;40(5):473–9. DOI: 10.1016/j.acalib.2014.06.003.

[22] Hider P, Pymm B. Empirical research methods reported in high-profile LIS journal literature. J Librariansh Inf Sci. 2008;30(2):108–14. DOI: 10.1016/j.lisr.2007.11.007.

[23] Koufogiannakis D, Slater L, Crumley E. A content analysis of librarianship research. J Info Sci. 2004;30(3):227–39. DOI: 10.1177/0165551504044668.

[24] The EQUATOR Network: Enhancing the QUAlity and Transparency Of Health Research [Internet]. EQUATOR Network [cited 2020 Dec 16]. Available from: https://www.equator-network.org/.

[25] Eldredge JD, Phillips HE, Kroth PJ. Real-time peer review: an innovative feature to an evidence-based practice conference. Med Ref Serv Q. 2013;32(4):412–23. DOI: 10.1080/02763869.2013.837690. 24180649 PMC3864097

