# Supplementary material for: The publication fate of abstracts presented at the Medical Library Association conferences
Source: J Med Libr Assoc. 2021 Oct 1;109(4):590–8. doi: 10.5195/jmla.2021.1220 (PMC8608162; doi:10.5195/jmla.2021.1220)
Supplement: Supplementary file 1 — Appendix A: Research method definitions [file jmla-109-4-590-s01.docx]

**Appendix A**

**Research Method Definitions**

| **Method** | **Definition** | **Example** |
| --- | --- | --- |
| Bibliometrics | Bibliometrics reveal patterns of scholarly communication, the development of a field of study, and impact factors of scholarly publications. | - Citation pattern study |
| Content analysis | Study of recorded communications to determine patterns, themes and infer meaning. | - Study examining content of websites, social media, books, articles, etc. |
| Experimental | Studies that explore relationships between independent and dependent variables and attempt to provide evidence of causal relationships. Typically use a randomized controlled or quasi-experimental (non-randomized) study design. | - Comparative study - Pre-post study (including educational interventions) - A-B study - Randomized controlled trial - Quasi-experimental trial |
| Focus groups/interview | Qualitative studies using focus groups or interviews |  |
| Mixed methods | Studies that use a combination of quantitative and qualitative methods |  |
| Observation/description/field study | Directed surveillance of an object or subject of investigation including the recording of observed data. Survey research, because of its high occurrence rate, has been placed in a separate category. | - Library space study - Systems analysis - Case study - Collection analysis |
| Secondary data analysis | Studies that reanalyze data from other sources. |  |
| Survey | Research based on data measured directly through questionnaires. Used to describe the characteristics of, and make predictions, about a population. |  |
| Literature review | In-depth study and evaluation of available information, usually in the form of a review or meta-analysis. | - Systematic review - Meta-analysis - Scoping review - Rapid review |
| Other methods | Use sparingly |  |

Definitions derived from these sources:

- Gore SA, Nordberg JM, Palmer LA, Piorun ME. Trends in health sciences library and information science research: an analysis of research publications in the Bulletin of the Medical Library Association and Journal of the Medical Library Association from 1991 to 2007. J Med Libr Assoc. 2009 Jul;97(3):203–11. Retrieved from <https://www.ncbi.nlm.nih.gov/pmc/articles/PMC2706445/>.
- Librarian and Research Knowledge Space (LARKS). Tools, Publications & Resources: Research Methods. 2014 [cited 2020 Dec 16]. Available from: <http://www.ala.org/tools/research/larks/researchmethods>.
