# Supplementary material for: The publication fate of abstracts presented at the Medical Library Association conferences
Source: J Med Libr Assoc. 2021 Oct 1;109(4):590–8. doi: 10.5195/jmla.2021.1220 (PMC8608162; doi:10.5195/jmla.2021.1220)
Supplement: Supplementary file 2 — Appendix B: Abstract data extraction instrument [file jmla-109-4-590-s02.docx]

**Appendix B**

**Abstract Data Extraction Instrument**

Q1 Reviewer

________________________________________________________________

Q2 Conference year

- 2012
- 2014

Q3 What is the format?

- Presentation
- Poster

Q4 Abstract ID

Format: PR for presentation/PO for poster, Number assigned to abstract

Example: PR221

________________________________________________________________

Q5 Presentation/Poster title. Write out exactly as written in the program.

________________________________________________________________

Q6 First author's full name. Write out exactly as written in the program, including middle initial. Do not add periods.

________________________________________________________________

Q7 Work setting of first author

- Hospital
- University or college
- Government or health association library
- Other ________________________________________________

Q8 Is there at least one author that is NOT from the United States according to their affiliation?

- Yes
- No

Q9 Is there at least one author that is NOT a librarian? Library science faculty, research faculty, and healthcare professionals count as non-librarians.

- Yes
- No

Q10 Are there authors from more than one institution?

- Yes
- No

Q11 Does the first author have the AHIP credential?

- Yes
- No

Q12 Is this a research study?

- Yes
- No

Q13 Add any notes/observations about whether or not this research here:

________________________________________________________________

*Display Question: If Is this a research study? = Yes*

Q14 What is the study design?

- Literature Review
- Descriptive
- Experimental
- Qualitative
- Mixed
- Other ________________________________________________

*Display This Question: If Is this a research study? = Yes*

Q15 Add any notes/observations about the study design here:

________________________________________________________________

*Display This Question: If Is this a research study? = Yes*

Q16 What research method does the study use?

- Bibliometrics
- Content analysis
- Experimental (random or non-random assignment. Includes pre/post educational interventions)
- Literature review (systematic, scoping, rapid, etc)
- Focus groups/interview
- Mixed methods (quantitative + qualitative methods)
- Observation/description/field study
- Secondary data analysis (re-use of dataset) (14)
- Survey
- Other methods (13) ________________________________________________

*Display This Question: If Is this a research study? = Yes*

Q17 Add any notes/observations about the research method here:

________________________________________________________________

Q18 What is the content domain?

- Information services
- Information management
- Education
- Management and Leadership
- Professionalism
- Other (non-library focused research)

Q19 Add any notes/observations about the content domain here:

________________________________________________________________

Q20 Was this abstract later published as a journal article?

Articles, commentaries, editorials, and letters count.

*Skip this question if you are not doing a search.

- Yes
- No

*Display This Question: If Was this abstract later published as a journal article? Articles, commentaries, editorials, and... = Yes*

Q21 Where was the abstract published? Write in full name of journal or other source.

________________________________________________________________

*Display This Question: If Was this abstract later published as a journal article? Articles, commentaries, editorials, and... = Yes*

Q22 What year was the abstract published? Format: YYYY

________________________________________________________________

*Display This Question: If Was this abstract later published as a journal article? Articles, commentaries, editorials, and... = Yes*

Q23 What month was the abstract published? Use numbers. Format: MM

________________________________________________________________

*Display This Question: If Was this abstract later published as a journal article? Articles, commentaries, editorials, and... = Yes*

Q24 Provide an identifier: DOI, PMID, URL

________________________________________________________________

Q25 Add any notes about the search here.

*Skip if not doing a search.

________________________________________________________________
