# Supplementary material for: The publication fate of abstracts presented at the Medical Library Association conferences
Source: J Med Libr Assoc. 2021 Oct 1;109(4):590–8. doi: 10.5195/jmla.2021.1220 (PMC8608162; doi:10.5195/jmla.2021.1220)
Supplement: Supplementary file 3 — Appendix C: Author questionnaire [file jmla-109-4-590-s03.docx]

**Appendix C**

**Author Questionnaire**

You are receiving this survey because you presented either a poster or paper at an annual meeting of the Medical Library Association (MLA) during 2012 or 2014.

This survey is part of MLA’s Research Training Institute and will determine the dissemination of research and projects presented at the annual conferences. This survey will take ten minutes or less. Your answers will be confidential. Survey responses will be stripped of personally identifiable information.

Please consider the list of your abstracts in the email sent to you when responding.

Thank you for your participation! If you need to reference the meeting programs, you can do so at MLA's website.

This survey was reviewed by the Indiana University Institutional Review Board and was declared to be exempt.

1. Did any of your MLA posters or presentations from 2012 and 2014 result in a published article or have been submitted publication?

- Yes
- No

*Skip To Q42: If Did any of your MLA posters or presentations from 2012 and 2014 result in a published article or... = No*

*Display This Question :If Did any of your MLA posters or presentations from 2012 and 2014 result in a published article or... = Yes*

2. Which abstract(s) has been published or submitted for publication? Note: There may be blank fields in this question.

▢ ${e://Field/Abstract1}

▢ ${e://Field/Abstract2}

▢ ${e://Field/Abstract3}

▢ ${e://Field/Abstract4}

*Display This Question: If Which abstract(s) has been published or submitted for publication? Note: There may be blank field... = ${e://Field/Abstract1}*

3. Where did you publish this abstract (${e://Field/Abstract1})? If you published in more than one source, consider the most recent publication.

- Peer-reviewed journal
- Non-peer-reviewed journal
- Other ________________________________________________
- I have submitted this abstract for publication.

*Display This Question:*

*If Where did you publish this abstract (${e://Field/Abstract1})? If you published in more than one s... = Peer-reviewed journal*

*Or Where did you publish this abstract (${e://Field/Abstract1})? If you published in more than one s... = Non-peer reviewed journal*

*Or Where did you publish this abstract (${e://Field/Abstract1})? If you published in more than one s... = Other*

4. Please provide the full name of the journal or other source that published your paper.

________________________________________________________________

5. What year was your paper published?

________________________________________________________________

*Display This Question: If Where did you publish this abstract (${e://Field/Abstract1})? If you published in more than one s... = I have submitted this abstract for publication.*

6. At what stage is your paper submission?

- Paper was accepted, publication pending. Please write in which journal accepted your paper: ________________________________________________
- Still awaiting a response from the editor.
- Paper was rejected.

*Display This Question: If Which abstract(s) has been published or submitted for publication? Note: There may be blank field... = ${e://Field/Abstract1}*

7. Select the primary reason you chose to pursue publication for this abstract: ${e://Field/Abstract1}

- To receive merit increments, tenure, and/or promotion due to my research activities
- Share results with a broader audience
- Contribute to my profession's evidence-base
- My research or project was novel
- I am (formally or informally) expected to participate in research as part of my job.
- My coauthors and/or colleagues encouraged me to publish
- To build a professional reputation for myself
- To demonstrate the impact of my library or the profession
- Other, please explain: ________________________________________________

8. What is the secondary reason you chose to pursue publication?

- To receive merit increments, tenure, and/or promotion due to my research activities
- Share results with a broader audience
- Contribute to my profession's evidence-base
- My research or project was novel
- I am (formally or informally) expected to participate in research as part of my job.
- My coauthors and/or colleagues encouraged me to publish
- To build a professional reputation for myself
- To demonstrate the impact of my library or the profession
- No other reason
- Other, please explain: ________________________________________________

**Note: These questions are then repeated for each abstract the participant said was published in question 2.**

Q42 What were your credentials at the time of the conference you presented at (2012 or 2014)? If you presented at both conferences, answer for 2014. Check all that apply.

- MLS or equivalent
- Non-MLS master's degree
- PhD
- Professional degree: MD, RD, RN, PA, PharmD, DO, etc.
- Other, please explain: ________________________________________________
- None of the above
